# Advanced Glycation End Products' Receptor DNA Methylation Associated with Immune Infiltration and Prognosis of Lung Adenocarcinoma and Lung Squamous Cell Carcinoma

**DOI:** 10.1155/2023/7129325

**Published:** 2023-07-18

**Authors:** Jun Yang, Mingqiang Lin, Mengyan Zhang, Zhiping Wang, Hancui Lin, Yilin Yu, Qunhao Zheng, Xiaohui Chen, Yahua Wu, Qiwei Yao, Jiancheng Li

**Affiliations:** ^1^Department of Radiation Oncology, Clinical Oncology School of Fujian Medical University, Fujian Cancer Hospital, No. 420 Fuma Rd., Jin'an District, Fuzhou 350014, Fujian, China; ^2^Clinical Oncology School of Fujian Medical University, No. 420 Fuma Rd., Jin'an District, Fuzhou 350014, Fujian, China; ^3^Department of Thoracic Surgery, Clinical Oncology School of Fujian Medical University, Fujian Cancer Hospital, No. 420 Fuma Rd., Jin'an District, Fuzhou 350014, Fujian, China

## Abstract

**Background:**

Advanced glycation end products' receptor (AGER) is a multiligand receptor that interacts with a wide range of ligands. Previous studies have shown that abnormal AGER expression is closely related to immune infiltration and tumorigenesis. However, the AGER DNA methylation relationship between prognosis and infiltrating immune cells in LUAD and LUSC is still unclear.

**Methods:**

AGER expression in pan-cancer was obtained by using the UALCAN databases. Kaplan–Meier plotter showed the correlation of *AGER* mRNA expression levels and clinicopathological parameters. The protein expression levels for AGER were derived from Human Protein Atlas Database Analysis. The copy number, somatic mutation, and DNA methylation of AGER were presented with UCSC Xena database. TIMER platform and TISIDB website were used to show the correlation between *AGER* expression and tumor immune cell infiltration level.

**Results:**

The expression level of *AGER* was significantly reduced in lung adenocarcinoma (LUAD) and lung squamous cell carcinoma (LUSC). Low expression of *AGER* was significantly correlated with histology, stage, lymph node metastasis, and tumor protein 53 (TP53) mutation and could be used as a potential indicator of poor prognosis of LUAD and LUSC. Moreover, *AGER* expression was positively correlated with the infiltrating immune cells. Further analysis showed that copy number variation (CNV), mutation, and DNA methylation were involved in AGER downregulation. In addition, we also found that hypermethylated AGER was significantly correlated with tumor-infiltrating lymphocytes.

**Conclusion:**

AGER may be a candidate for the prognostic biomarker of LUAD and LUSC related to tumor immune microenvironment.

## 1. Introduction

Cancer as a major public health problem, the morbidity and mortality have risen sharply worldwide, placing a heavy burden on the public health system. In 91 of 172 countries, cancer is the first or second leading cause of death before age 70 [1–3]. Due to the typical early clinical symptoms that are not obvious and the limitations of diagnostic methods, the vast majority of patients with lung cancer is diagnosed at a later stage [4]. Over the past few decades, thanks to the efforts of clinical and scientific researchers, breakthroughs have been made in the diagnosis and treatment of lung cancer [5, 6]. Therefore, the 5-year survival rate of patients diagnosed with lung cancer is not satisfactory, only an astonishing 15%, while the prognosis of individuals diagnosed with advanced disease is even worse [7]. Hence, screening for potential lung cancer gene therapy targets and prognostic markers is particularly important.

Advanced glycation end products (AGEs) refer to a group of heterogeneous macromolecules that are produced by post-translational modification of proteins through nonenzymatic glycation, lipids, aging, and nucleic acids [8]. AGEs provide the bridge between intracellular and extracellular damage through the advanced glycation end products' receptor (AGER), also known as the receptor for advanced glycation end products (RAGE). AGER protein is a multiligand receptor that interacts with a wide range of ligands, including AGEs, *β*-sheet fibrils, S100 proteins (S100B, S100P, S100A4, S100A6, S100A8/9, and S100A11–13), high mobility family protein-1, and prion [9, 10]. AGER expression plays a central role in the neurodegeneration, retinal microvascular dysfunction, and thymic hyperplasia via the toll-like receptor 4 and AGE/AGER signaling pathways [10]. Nevertheless, AGER expression can be induced under certain pathological conditions (including high glucose, reactive oxygen species, hypoxia, proinflammatory mediators, or AGER itself) [8, 10].

Tumor microenvironment (TME) comprises a complex milieu of nonmalignant cells including vascular vessels, fibroblasts, extracellular matrix, and immune infiltrates, which can interact closely with tumor cells and affect tumor growth and metastasis [11]. Immune infiltration plays a central role in the tumor microenvironment, especially tumor-infiltrating lymphocytes [12]. Previous studies have shown that abnormal AGER expression is closely related to immune inflammatory response and tumorigenesis [13]. Other related studies also showed that AGER expression and mutation play an important role in brain disease, esophageal cancer, breast cancer, gastric cancer, prostate, melanoma, and endometrial carcinoma [14–24]. Some reports have also been made on AGER in non-small-cell lung cancer (NSCLC). Excellent studies have shown that low expression of AGER significantly reduced the median survival time of LUAD patients [25, 26]. To clarify the mechanism of action between AGER in NSCLC, Yang et al. [27] verified the function of AGER in modulating the tumor microenvironment via miR-182-5p/NF-*κ*B axis mediating the malignant phenotypes of NSCLC. During the occurrence and metastasis of lung cancer, AGER‘s significance has been demonstrated in the progression, angiogenesis, and immune cell infiltration mediated by lysophosphatidic acid [28]. Although AGER has multitudinous functions in the tumor microenvironment, numerous mechanisms are still unclear, especially the potential mechanism between DNA methylation and lymphocyte infiltration in LUAD and LUSC.

In our work, Tumor Immune Estimation Resource (TIMER), Gene Expression Profiling Interactive Analysis (GEPIA), UALCAN, and Kaplan–Meier plotter databases were used to demonstrate *AGER* expression level and its correlation with the prognosis. Furthermore, we used the TIMER network resource to explicate the associations of *AGER* and important components of the tumor microenvironment (tumor-infiltrating immune cells). We also explained the relativity between tumor-infiltrating immune cells and prognosis. In addition, we further explored the potential molecular mechanism of *AGER* imbalance including CNV, somatic mutation, and DNA methylation. Furthermore, we clarified that the high degree of *AGER* DNA methylation was obviously related to infiltrating lymphocytes. Thus, we raise a possible regulatory mechanism of *AGER* DNA methylation and tumor-infiltrating lymphocytes which influence prognoses of LUAD and LUSC to some extent.

## 2. Materials and Methods

### 2.1. Tumor Immune Estimation Resource (TIMER) Database Analysis

The TIMER database is a feature-rich resource. The TIMER algorithm is used to systematically analyze the relationship between gene expression of different cancer types and tumor-infiltrating immune cells. The abundance of six tumor-infiltrating cells was assessed [29]. The TIMER website is used to illustrate the differential expression of AGER in normal and tumor tissues in diverse malignant tumors. Moreover, we analyzed the relationship between *AGER* and 6 types of tumor-infiltrating immune cells in the “Gene” module. We also used this site to investigate the relationship between gene expression level and immune-infiltrating cells in LUAD and LUSC.

### 2.2. Gene Expression Profiling Interactive Analysis (GEPIA)

GEPIA is a newly developed database that provides customizable functions with RNA sequencing expression data form 9736 tumors and 8587 normal samples. It is a useful network resource for visualization of gene expression based on The Cancer Genome Atlas (TCGA) and Genotype Tissue Expression (GTEx) data [30]. We showed various expression levels of *AGER* in normal and tumor tissues in different tumors. In LUAD and LUSC, normal and tumor tissues were used to detect the expression level of *AGER*. In addition, the survival module contributes to clarify the relationship between *AGER* expression and prognosis.

### 2.3. Human Protein Atlas Database Analysis

The Human Protein Atlas is an efficient and open database that allows free access by academic researchers and provides a reference for exploring the human proteome [31, 32]. We focused on Pathology Atlas, which shows the impact of protein levels for the survival of patients with cancer. We screened protein expression in LUAD and LUSC through immunohistochemistry in the pathology module.

### 2.4. UALCAN Database Analysis

UALCAN is a fully functional, friendly, and interactive network resource, mainly used to analyze cancer omics data. By linking multiple databases, the expression analysis of genes, proteins, and epigenetics can be quickly realized. These resources enable researchers to efficiently lock interesting targets and valuable information [33]. We used the UALCAN web resource to verify the results between *AGER* and various clinicopathological parameters including pathology, cancer stages, nodal metastasis status, and TP53 mutation status of lung cancer and calculated the *P* value.

### 2.5. Kaplan–Meier Plotter Database Analysis

Kaplan–Meier plotter downloads gene expression data, recurrence-free, and overall survival information through links to GEO, EGA, and TCGA and then meta-analyzes the prognostic value of a specific gene. Its database has been able to assess the impact of more than 50,000 genes (mRNA, miRNA, and protein) on the survival rate of 21 cancer types and is a commonly used tool for bioinformatics analysis [34]. Kaplan–Meier plotter web resources were used to verify the correlation between diverse clinical results and the expression of *AGER* in LUAD and LUSC. We showed a prognostic analysis of *AGER* expression in distinct immune cell subsets with this web.

### 2.6. PrognoScan Database Analysis

The PrognoScan database is a publicly available cancer microarray dataset with clinical annotation function, which can be used as an online analysis tool to evaluate the biological relationship between gene expression and prognosis. A systematic meta-analysis can be performed on multiple datasets. It is a powerful platform for evaluating potential tumor markers and treatment targets. Its existence will certainly promote cancer research [35]. This database was used to illustrate the effects of abnormal *AGER* expression on the prognosis in lung cancer, LUAD, and LUSC.

### 2.7. TISIDB Database Analysis

TISIDB is an open, free, and useful database. It integrates data from multiple public databases including UniProt, Gene Ontology (GO), DrugBank, PubMed, and TCGA. It aims to clarify the interaction between tumors and immune cells and is a valuable resource for cancer immunology research and treatment [36]. To illustrate the potential relationship between *AGER* and tumor-infiltrating lymphocytes (TILs), 28 TILs were used to analyze the association with *AGER* in different tumor sites in the TISIDB database. Besides, we also demonstrated the correlation between AGER DNA methylation and tumor-infiltrating lymphocytes via this platform.

### 2.8. UCSC Xena Database Analysis

UCSC Xena database provides interactive online visualization of seed cancer genomics datasets, which can support online analysis of a variety of genomics, proteomics, phenotype, and clinical annotation data. It has included more than 50 cancer-type related data and is a user-friendly database [37]. In the study, gene expression, copy number, somatic mutation, and DNA methylation were presented in this database. Details of the probe cohorts for detecting AGER DNA methylation and the level of methylation are also displayed.

### 2.9. Statistical Analysis

TIMER, Kaplan–Meier plotter, PrognoScan, GEPIA, and UALCAN network resources were used for AGER expression verification. The survival curve based on the Kaplan–Meier plotter and GEPIA was presented using HR and *P* or *P* values from a log-rank test. SPSS 25.0 (SPSS, Inc., Chicago, IL) was used for data analysis. For two-group comparison, Student's *t*-test method was used. Two-tailed *P* < 0.05 was considered statistically significant.

## 3. Results

### 3.1. *AGER* Expression Level Is Downregulated in LUAD and LUSC Patients

**Figure 1:**
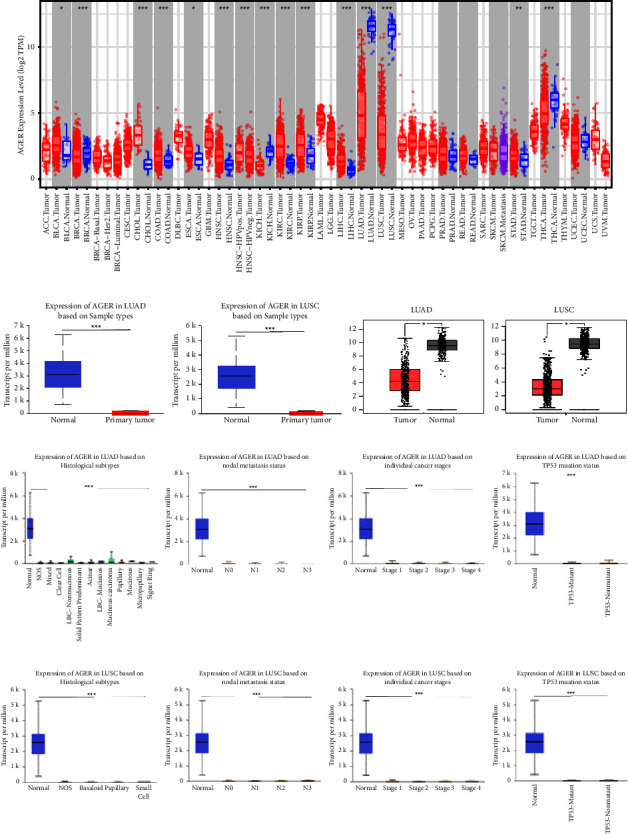
The expression of *AGER* in pan-cancer and correlation with different clinicopathological features in LUAD and LUSC: (a) the expression of *AGER* in pan-cancer in the TIMER online resource, (b) *AGER* mRNA expression level in LUAD/LUSC and normal patients in the UALCAN (left) and GEPIA databases (right), (c) the expression of *AGER* in various clinicopathological features (tumor histology, stage, lymph node metastasis, and TP53 mutation) in LUAD, and (d) expression of *AGER* in different clinicopathological features (tumor histology, stage, lymph node metastasis, and TP53 mutation) in LUSC. ^*∗*^*P* < 0.05, ^*∗∗*^*P* < 0.01, and ^*∗∗∗*^*P* < 0.001.

The *AGER* expression level in different cancer types was elaborated using the TIMER web database. Lower expression of *AGER* was revealed in breast invasive carcinoma (BRCA), thyroid carcinoma (THCA), kidney chromophobe (KICH), LUAD, and LUSC compared with corresponding normal tissues. On the contrary, in bladder urothelial carcinoma (BLCA), cholangiocarcinoma (CHOL), esophageal carcinoma (ESCA), head and neck squamous cell carcinoma (HNSC), kidney renal clear cell carcinoma (KIRC), kidney renal papillary cell carcinoma (KIRP), liver hepatocellular carcinoma (LIHC), and stomach adenocarcinoma (STAD) compared with the control group, *AGER* showed a high expression trend (Figure 1(a)). In the UALCAN database, the result showed that the expression level of AGER in the normal lung tissue was significantly higher than that in LUAD and LUSC (Figure 1(b)). This result indicates that AGER may act as a significant part in the biological process of lung cancer.

Furthermore, the expression level of *AGER* in lung cancer samples and adjacent tissues is obtained from GEPIA online resource. *AGER* expression was significantly decreased in LUAD and LUSC (Figure 1(b)). The same result was also verified in the UALCAN database. Further studies showed that the expression of *AGER* in tumor histology, stage, lymph node metastasis, and TP53 mutation was significantly increased in normal tissues, and it was low in LUAD and LUSC tumor tissues (Figures 1(c) and 1(d)).

### 3.2. AGER Protein Presented Low Expression in LUAD and LUSC Tissues

**Figure 2:**
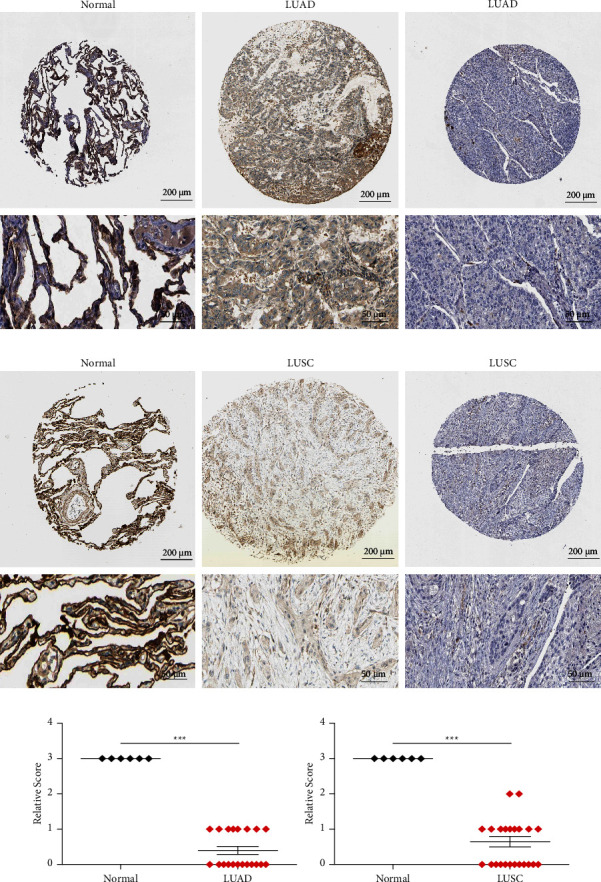
The expression level of AGER protein in LUAD and LUSC tissues. (a) In LUAD, the protein expression level of AGER in the Human Protein Atlas database. (b) In LUSC, the protein expression level of AGER according to the Human Protein Atlas database. (c) LUAD and LUSC were quantitatively analyzed with GraphPad Prism 5.0. Scale: 200 *μ*m (upper) and 50 *μ*m (lower). ^*∗∗∗*^*P* < 0.001.

Protein expression levels in LUAD and LUSC obtained and visualized with the Human Protein Atlas database. We then established a scoring system whereby high levels of positive AGER expression received 3 points, moderate levels received 2 points, low levels received 1 point, and no expression received 0 points. The results indicated that AGER exhibited moderate positive expression in all 4 normal tissues. In addition, there were 0, 0, 8, and 12 cases of high, medium, low levels positive, and undetected staining in LUAD and 0, 2, 9, and 9 cases in LUSC (Figures 2(a) and 2(b)), respectively. It was observed that in both LUAD and LUSC, the expression of AGER was significantly decreased in tumor tissues, as shown in Figure 2(c).

### 3.3. The Prognostic Value of *AGER* Was Verified Based on Kinds of Clinicopathological Features

**Figure 3:**
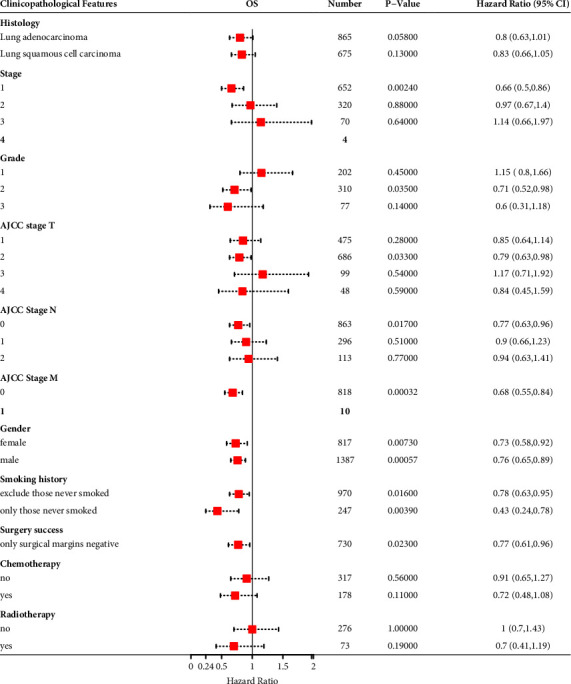
Forest plot of prognostic value of AGER in different clinicopathological features.

To understand the relationship between AGER and prognostic value in more detail, we investigated the correlation between *AGER* mRNA expression and clinicopathological features using Kaplan–Meier database. Interestingly, low expression *AGER* was associated with poor overall survive (OS) only in American Joint Committee on Cancer (AJCC) stage T2 of lung cancer patients (Figure 3). Then, the correlation between *AGER* expression and poor OS was observed in AJCC stage N0 population (Figure 3). Then, low *AGER* expression was evidently associated with poor OS in both males and females (Figure 3). Moreover, we clarified that low expression *AGER* represented worse OS in both smoking and nonsmoking patients (Figure 3). Then, low *AGER* expression was obviously related with poor OS in lung cancer patients with negative surgical margins (Figure 3). Furthermore, we observed that in patients who received chemotherapy or radiotherapy, low level of AGER indicated worse OS, but without statistical significance (Figure 3). These results indicate that the prognostic value of low expression of AGER for lung cancer is meaningful.

### 3.4. Lower *AGER* Expression Is Related to Poor Prognosis in Lung Cancer Patients

**Figure 4:**
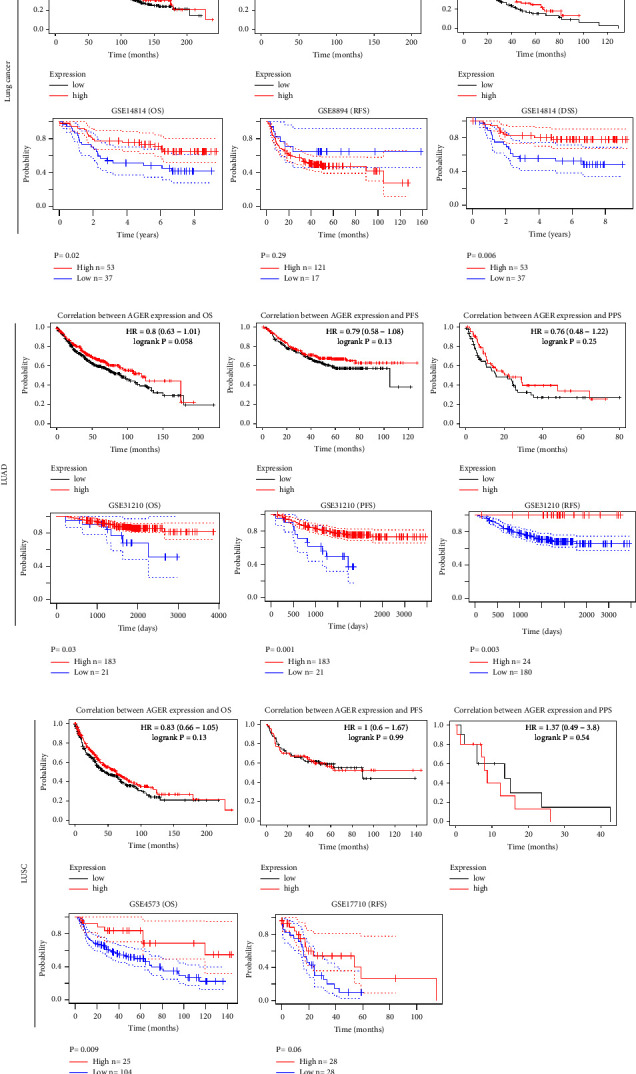
Survival curve assesses the prognostic value of *AGER* in lung cancer, LUAD, and LUSC. The OS, PFS, and PPS of lung cancer (a), LUAD (b), and LUSC (c) survival curves were displayed using the Kaplan–Meier plotter database (upper). The OS, RFS, DSS, and PFS of lung cancer (a), LUAD (b), and LUSC (c) survival curves were shown using the PrognoScan database (lower).

We investigated the Kaplan–Meier plotter and PrognoScan database for the prognostic feature of *AGER* expression in lung cancer. Lower expression of the *AGER* gene showed worse OS, progression-free survival (PFS), and postprogression survival (PPS) based on the Kaplan–Meier plotter database (Figure 4(a) upper). The other side of the shield, the PrognoScan database, presented that decreased expression of *AGER* represented a poor OS in the GSE14814 and disease-specific survival (DSS) in the GSE14814 cohorts but not in relapse-free survival (RFS) in the GSE8894 (Figure 4(a) lower). These results indicate that *AGER* is noticeably associated with the prognosis of lung cancer patients. However, in further analysis, it is found that the conclusions drawn by the two databases are not completely consistent. In PrognoScan database, both LUAD and LUSC with low expression of *AGER* were associated with poor OS, PFS, and RFS (Figures 4(b) and 4(c)). However, the abovementioned conclusions were not reached in the Kaplan–Meier plotter database, especially in LUSC where relevant prognostic indicators such as OS, PFS, and PPS failed to support the same conclusion (Figure 4(c)).

### 3.5. Relativity Analysis between Low Level *AGER* and Infiltrating Immune Cells in LUAD and LUSC

**Figure 5:**
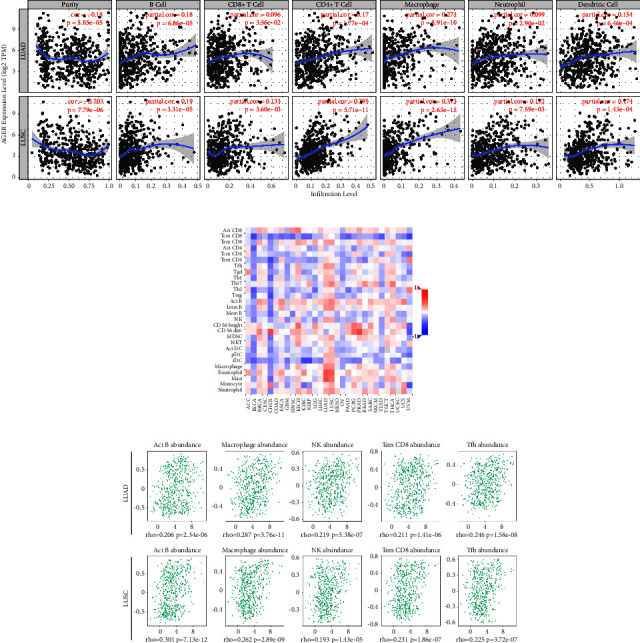
Correlation analysis of *AGER* expression and tumor immune infiltration in LUAD and LUSC: (a) *AGER* expression in LUAD and LUSC obviously correlated with tumor immune infiltration, (b) correlation between *AGER* expression and 28 tumor-infiltrating lymphocytes in pan-cancer, and (c) the top 5 immune-infiltrating lymphocytes significantly correlated with *AGER* expression in LUAD and LUSC.

Tumor-infiltrating immune cells can be used independently to predict the status of tumor sentinel lymph node metastasis and prognosis [38]. We elaborated the correlation between *AGER* expression and 6 types of immune cells which is default in the database, including B cells, CD4^+^ T cells, CD8^+^ T cells, neutrophils, macrophages, and dendritic cells with TIMER database. The results showed that *AGER* correlated with infiltration of B cells, CD4^+^ T cells, CD8^+^ T cells, neutrophils, macrophages, and dendritic cells. In addition, both LUAD and LUSC reached the same conclusion (Figure 5(a)).

We investigated the correlation between *AGER* expression level and 28 tumor immune infiltrating lymphocyte subtypes. Those results demonstrated that *AGER* was linked to 21 and 20 different lymphocyte subtypes in LUAD and LUSC, respectively (Figure 5(b) and Table 1). Especially, it is significantly related to activated B cell, macrophage, natural killer cell, effector memory, CD8^+^ T cell, and T follicular helper cell, both in LUAD and LUSC (Figure 5(c)).

### 3.6. Prognostic Value of *AGER* Expression in LUAD and LUSC Based on Diverse Immune Cells

**Figure 6:**
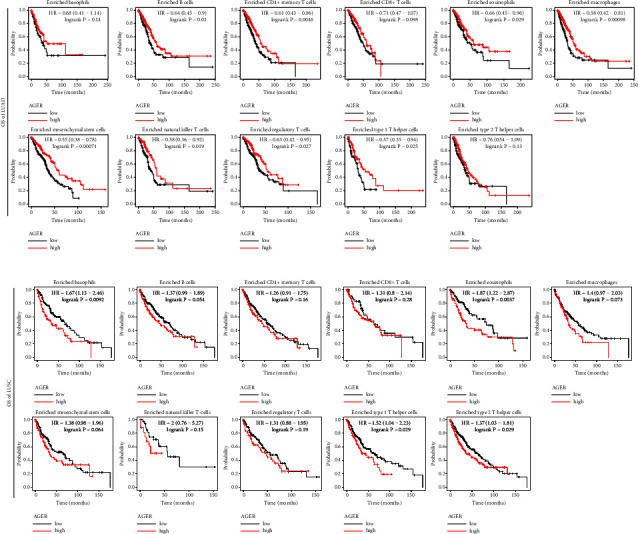
Correlation between expression level of *AGER* and prognosis in LUAD and LUSC with different subtypes of infiltrating immune cells. The Kaplan–Meier plotter database revealed the relationship between *AGER* and OS in different subtypes of infiltrating immune cells in LUAD (a) and LUSC (b).

Our research indicated *AGER* expression related to the immune infiltration of LUAD and LUSC. In addition, low level of *AGER* was involved with the poor prognosis of lung cancer. Therefore, we intended to investigate whether AGER might impact the prognosis of LUAD and LUSC through immune infiltration to some extent. We reported that LUAD patients with low AGER levels in enriched B cells, CD4^+^ memory T cells, eosinophils, macrophages, mesenchymal stem cells, natural killer T cells, regulatory T cells, and type 1 T helper cells had poor prognosis (Figure 6(a)). Interestingly, the high *AGER* level in LUSC-enriched Basophils, Eosinophils, macrophages, Type 1 T helper cells, and Type 2 T helper cells cohort had a worse prognosis (Figure 6(b)). The data suggest that different expression levels of AGER may affect the immune infiltration cells of diverse subtypes of lung cancer, such as LUAD and LUSC, ultimately influencing their prognosis.

### 3.7. CNV, Mutation, and DNA Methylation Analysis of *AGER* Gene in LUAD and LUSC

**Figure 7:**
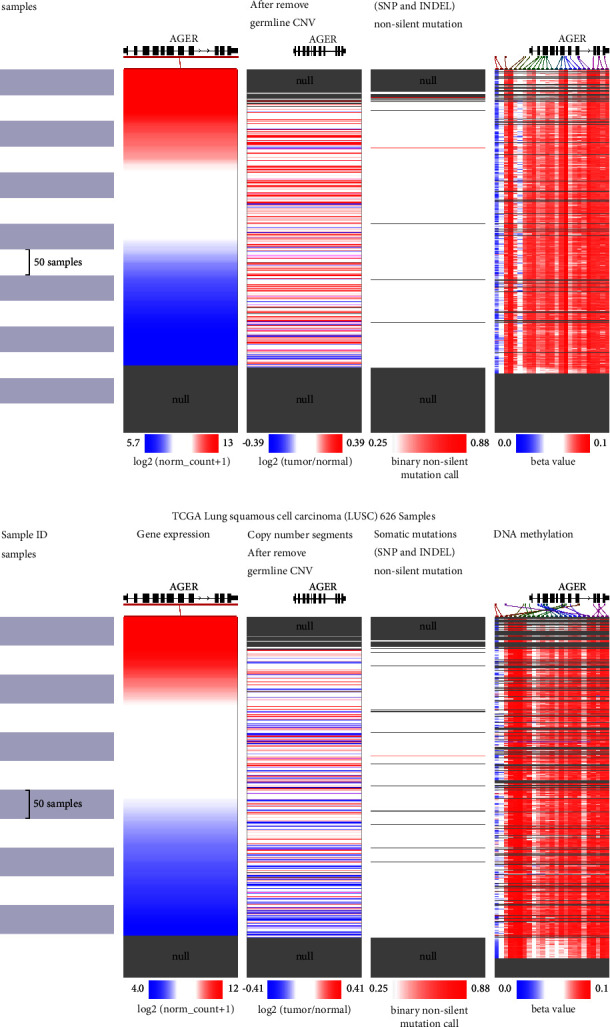
CNV, mutation, and DNA methylation analysis of *AGER* gene in LUAD and LUSC. Heatmaps displayed the relation between *AGER* mRNA and CNV, somatic mutations, and DNA methylation in LUAD (a) and LUSC (b).

We further explored the expression, CNV, gene mutation, and DNA methylation levels of AGER in LUAD and LUSC through UCSC Xena database. Heatmap analysis revealed a correlation between *AGER* mRNA expression and CNV and gene mutation and DNA methylation in LUAD (Figure 7(a)) and LUSC (Figure 7(b)). Simultaneously, the heatmap also indicated that *AGER* DNA methylation levels in LUAD and LUSC were higher than normal tissues (Figures 7(a) and 7(b)).

### 3.8. AGER DNA Methylation Was Obviously Related to Tumor Immune Infiltrating Lymphocyte Subtypes

**Figure 8:**
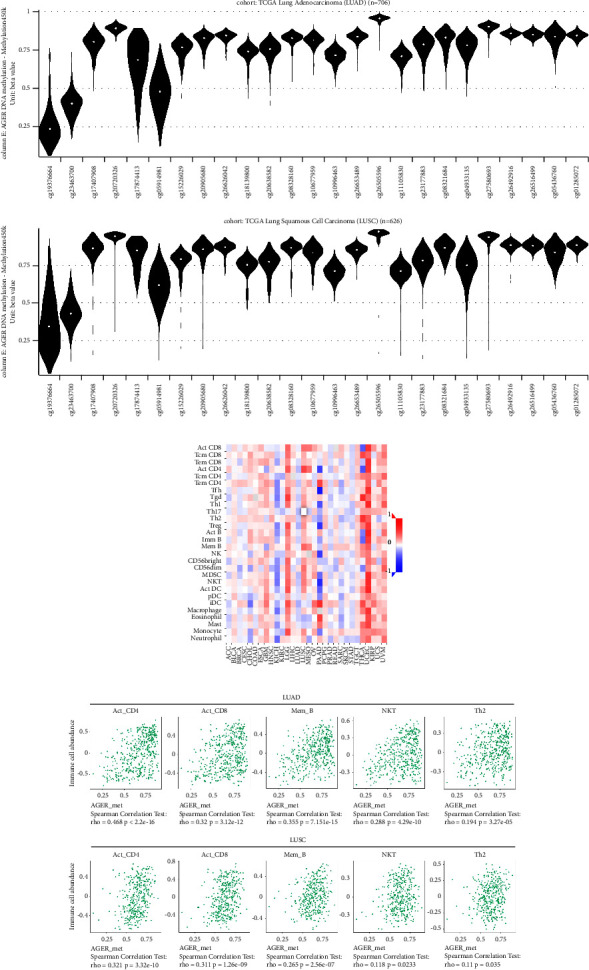
The correlation between AGER DNA methylation and tumor immune-infiltrating lymphocyte subtypes in LUAD and LUSC: (a) the signal intensity of AGER DNA methylation detected by different probe cohorts, (b) relevance between AGER DNA methylation and 28 tumor immune-infiltrating lymphocyte subtypes in pan-cancer, and (c) the top 5 immune-infiltrating lymphocyte subtypes notably related to AGER DNA methylation in LUAD and LUSC.

We have clarified that AGER displays high level of DNA methylation in LUAD and LUSC. We utilized UCSC Xena to establish the correlation between AGER DNA methylation and immune-infiltrating lymphocytes. The signal intensity of DNA methylation is detected by various probe cohorts and then expressed in the form of *β* value. Any *β* value of 0.6 or higher is considered fully methylated, while *β* value of 0.2 or lower is considered completely unmethylated. A *β* value between 0.2 and 0.6 is partially methylated [39]. In LUAD, out of the 25 probes, complete DNA methylation was observed in 22 probes while 3 showed partial DNA methylation (Figure 8(a) upper). Consistently, 23 probes were detected in LUSC suggesting complete DNA methylation, while 2 showed partial DNA methylation (Figure 8(a) lower). TISIDB was utilized to further investigate the relationship between AGER and tumor-infiltrating lymphocytes. Those results exhibited that it was significantly related to active CD4 cells, active CD8 cells, memory B cells, natural killer T cells, and type 2 T helper cells both in LUAD and LUSC (Figure 8(c)). This indicates a possible association between the DNA methylation of AGER and tumor-infiltrating immune lymphocytes.

## 4. Discussion

In recent decades, lung cancer has emerged as the primary cause of cancer-related deaths on a worldwide scale. Lung cancer is divided into non-small-cell lung cancer and small cell lung cancer according to the pathological type. Among them, NSCLC accounts for 85% of all lung cancer [1, 3, 40]. Therefore, it is imperative to focus on improving the level of diagnosis and treatment of NSCLC. Despite the promising results of immune checkpoint inhibitors in the treatment of lung cancer, the efficacy has not matched the anticipated outcomes [41]. Thus, it is essential to explore the mechanism of immunotherapy and identify promising prognostic biomarkers for lung cancer. Our research suggested that the expression of *AGER* was significantly downregulated in LUAD and LUSC using bioinformatics analysis of GEPIA, TIMER, and UALCAN databases (Figure 1(a)). At the same time, the protein level has also been further verified. Consistent with the conclusion reached at the gene level that *AGER* has lower expression in LUAD and LUSC (Figures 2(a) and 2(b)). These results were aggregated into valuable information and further showed that AGER may play the role of tumor suppressor involved in the occurrence of lung cancer. Then, the clinical prognostic significance of *AGER* in patients with LUAD and LUSC was reported. The downregulation of *AGER* was significantly correlated with tumor histology, stage, lymph node metastasis, and TP53 mutation of LUAD and LUSC patients (Figures 1(c) and 1(d)). In addition, Kaplan–Meier survival analysis presented that overexpression *AGER* was notable live longer than those patients with low AGER expression (Figure 4). Hence, AGER has the potential to serve as a valuable prognostic biomarker for patients with NSCLC.

Increasingly substantial evidence demonstrates that LUAD and LUSC exhibit distinguishable characteristics in numerous aspects, comprising gene expression profile, biological behavior, molecular pathological features, clinical features, and therapeutic responses [42]. Compared with LUAD, LUSC is usually associated with smoking and inflammatory diseases. Generally speaking, LUSC grows more slowly than LUAD during the same period, and the volume of the mass is smaller, but most patients have the tendency of early metastasis [43]. There are also significant differences between LUAD and LUSC in the gene mutation spectrum. Previous reports indicated that mutations of epidermal growth factor receptor (EGFR) gene are the commonest type of NSCLC patients. The frequency of EGFR mutations is 27% and 9% in LUAD and LUSC, respectively [44]. In addition, studies have shown that there are also great differences in mRNA, protein expression, signal transduction pathway, and DNA methylation mode between LUAD and LUSC [45–47]. These findings provide valuable experience and research basis for explaining the molecular mechanism of LUAD and LUSC. Similarly, in the research, we also found that AGER had differences in gene, protein level, and prognosis in LUAD and LUSC. In terms of protein expression level, *AGER* in LUSC was higher than that in LUAD (Figure 2). This also proved that low expression of *AGER* was related to a worse prognosis, consistent with previous research outcomes. In addition, we only observe that low expression *AGER* was related to OS in LUAD, but not in LUSC (Figure 3). This may be related to the different datasets selected by the database for analysis. Moreover, a limited number of samples may also have contributed to bias in the results. This also further verified the heterogeneity of LUAD and LUSC.

Immune cells have irreplaceable involvement in cancer progression and aggressiveness [48]. It is considered to be an important determinant of prognosis and the efficacy of immunotherapy [49]. In previous meaningful studies, immunohistochemical experiments showed that downregulating the AGER could significantly upregulate angiogenesis (CD34), leukocyte (CD45), and macrophage (F4/80) markers level. Further research pointed out that lysophosphatidic acid (LPA) induces proliferation, migration, colonization, and tumor microenvironment via RAGE and downstream protein kinase B (PKB) pathways [28]. In nontumor studies, it has been confirmed that AGER interacts with immune cells [50]. Valuable study had pointed out that in diabetic mouse models, RAGE was involved in tissue repair related to inflammatory damage. In-depth study has shown that RAGE downregulates the expression of pro-repair inflammatory genes in ischemic muscle and lowers the number of macrophages [51]. Similarly, we reported that low expression levels of *AGER* in LUAD and LUSC were linked to reduced infiltration of B cells, CD4^+^ T cells, CD8^+^ T cells, neutrophils, macrophages, and dendritic cells (Figure 5(a)). Moreover, we disclosed the correlation analysis between *AGER* and 28 tumor-infiltrating lymphocytes (Figure 5(b) and Table 1). It should be emphasized that it was significantly related to activated B cell, macrophage, natural killer cell, effector memory, CD8^+^ T cell, and T follicular helper cell, both in LUAD and LUSC (Figure 5(c)). Moreover, AGER has a partial impact on the survival time of LUAD and LUSC patients by immune cell infiltration (Figures 5(a) and 5(b)). This indicates that AGER could potentially be targeted for immune-related therapy in cases of lung cancer.

Epigenetics plays a key role in the regulation of gene expression [52]. Epigenetic regulation of genes can enable organisms to quickly adapt to changes in the new environment to obtain characteristics that are beneficial to themselves. It should be noted that epigenetic disorders can trigger the repression of tumor suppressor genes or the stimulation of oncogenes, ultimately serving as a contributing factor to tumor development and progression. As a common epigenetic phenomenon of tumors, DNA methylation features can be used as biomarkers for the prognosis and diagnosis of different cancer types and provide more optimized strategies for cancer treatment. The medical benefits of it are gaining broad recognition [53]. In our research, we discovered that *AGER* expression exhibited strong correlation with CNV, somatic mutations, and DNA methylation (Figures 6(a) and 6(b)). Furthermore, we clarified *AGER* was related to the tumor-infiltrating lymphocytes of LUAD and LUSC patients. In addition, our subsequent investigation revealed significant abnormalities in the DNA methylation status of these two types of cancers. This leads us to propose a hypothesis whether there is a mutual regulatory relationship between tumor-infiltrating lymphocytes and DNA methylation. Subsequent studies showed that highly DNA-methylated AGER in LUAD was closely correlated with active CD4 cells, active CD8 cells, memory B cells, natural killer T cells, and type 2 T helper cells. Similar phenomena could also be observed in LUSC. However, the potential mechanism of tumor immune microenvironment and AGER DNA methylation still needs to be further investigated. As for the relevance between AGER and lung cancer, this study has provided a new vision and expanded our understanding of the mechanisms that contribute to the development of lung cancer. However, it is undeniable that this study also has some limitations. Firstly, we focused on LUAD and LUSC in NSCLC, while SCLC, lung sarcoma, and other types of tumors were not involved. In fact, different pathological types of tumors exhibit significant variations in biological behavior and prognosis. Secondly, infiltrating immune cells, a major participant in the tumor microenvironment, have a variety of types and complex mechanisms. Thus, a hierarchical analysis is required to thoroughly explore their functions. Overall, the downregulation of AGER implies the critical role in the occurrence and development of lung cancer.

## 5. Conclusion

The gene and protein expression of AGER in LUAD and LUSC was downregulated, and it was obviously related to the prognosis. After adjusted by tumor purity, AGER showed a significant association with the tumor-infiltrating lymphocytes. Further analysis showed that AGER DNA methylation may be correlated with tumor-infiltrating lymphocytes, especially CD4^+^ T cells, active CD8^+^ T cells, memory B cells, natural killer T cells, and type 2 T helper cells. Consequently, our study provides insight into a novel role of AGER expression and DNA methylation in tumor immune infiltration. AGER could be a potential prognostic biomarker of LUAD and LUSC related to tumor-infiltrating lymphocytes. It should be noted that we recognize the following limitations in our research: our research findings were primarily obtained through bioinformatics analysis, without undergoing any additional experimental validation. Data obtained from different laboratories, platforms, and equipment may exhibit some variations. Numerous databases lack a consistent standard for integrating data and ensuring data quality during collection, resulting in the presence of bias. In fact, the database adopts strict standards and reasonable algorithms when incorporating relevant data to maximize the availability of data. In addition, our results are primarily based on bioinformatics analysis and have not been subjected to additional experimental validation. However, the data in the database are also compiled and analyzed based on the collection of clinical samples and various clinicopathologic features, providing a certain reference value. Furthermore, due to influences such as gene regulation and gene interactions, there are complex and intricate interactions between molecules and cells within organisms. Our results only indicate a correlation between AGER and other clinical pathological features but do not elucidate its regulatory relationship. Further exploration can be conducted through additional experiments to establish specific regulatory mechanisms. We need to recognize the limitations of bioinformatics analysis clearly, which is a prerequisite for improving the efficiency and accuracy of our research.

## Figures and Tables

**Table 1:** The correlation between AGER expression and tumor lymphocyte infiltration in LUAD and LUSC.

	LUAD		LUSC	
	*r*	*P*	*r*	*P*
Activated CD8 T cell (Act_CD8)	0.027	0.533	0.038	0.398
Central memory CD8 T cell (Tcm_CD8)	0.08	0.0708	−0.001	0.976
Effector memory CD8 T cell (Tem_CD8)	0.211	^*∗∗∗*^	0.231	^*∗∗∗*^
Activated CD4 T cell (Act_CD4)	−0.146	^*∗∗∗*^	−0.041	0.36
Central memory CD4T cell (Tcm_CD4)	0.027	0.537	0.037	0.413
Effector memory CD4 T cell (Tem_CD4)	0.113	^*∗∗*^	0.031	0.491
T follicular helper cell (Tfh)	0.246	^*∗∗∗*^	0.225	^*∗∗∗*^
Gamma delta T cell (Tgd)	0.121	^*∗∗*^	0.093	^*∗*^
Type 1 T helper cell (Th1)	0.168	^*∗∗∗*^	0.155	^*∗∗∗*^
Type 17 T helper cell (Th17)	0.192	^*∗∗∗*^	0.301	^*∗∗∗*^
Type 2 T helper cell (Th2)	0.045	0.311	0.046	0.3
Regulatory T cell (Treg)	0.11	^*∗*^	0.152	^*∗∗∗*^
Activated B cell (Act_B)	0.206	^*∗∗∗*^	0.301	^*∗∗∗*^
Immature B cell (Imm_B)	0.25	^*∗∗∗*^	0.27	^*∗∗∗*^
Memory B cell (Mem_B)	−0.055	0.208	−0.059	0.187
Natural killer cell (NK)	0.219	^*∗∗∗*^	−0.193	^*∗∗∗*^
CD56bright natural killer cell (CD56bright)	0.001	0.982	−0.027	0.54
CD56dim natural killer cell (CD56dim)	−0.185	^*∗∗∗*^	0.021	0.642
Myeloid derived suppressor cell (MDSC)	0.12	^*∗∗*^	0.206	^*∗∗∗*^
Natural killer T cell (NKT)	0.102	^*∗*^	0.079	0.0783
Activated dendritic cell (Act_DC)	0.062	0.157	0.164	^*∗∗∗*^
Plasmacytoid dendritic cell (pDC)	0.177	^*∗∗∗*^	0.169	^*∗∗∗*^
Immature dendritic cell (iDC)	0.138	^*∗∗*^	0.161	^*∗∗∗*^
Macrophage	0.287	^*∗∗∗*^	0.262	^*∗∗∗*^
Eosinophil	0.496	^*∗∗∗*^	0.432	^*∗∗∗*^
Mast cell	0.479	^*∗∗∗*^	0.368	^*∗∗∗*^
Monocyte	0.087	^*∗*^	0.204	^*∗∗∗*^
Neutrophil	0.203	^*∗∗∗*^	0.272	^*∗∗∗*^

^*∗*^ *P* < 0.05, ^*∗∗*^*P* < 0.01, and ^*∗∗∗*^*P* < 0.001.

## Data Availability

The data used to support the findings of this study are available from the corresponding author upon request.

## Abbreviations

AGER: Advanced glycation end products' receptor
AGEs: Advanced glycation end products
AJCC: American Joint Committee on Cancer
BRCA: Breast invasive carcinoma
BLCA: Bladder urothelial carcinoma
CNV: Copy number variation
CHOL: Cholangiocarcinoma
DSS: Disease-specific survival
ESCA: Esophageal carcinoma
EGFR: Epidermal growth factor receptor
GEPIA: Gene expression profiling interactive analysis
GTEx: Genotype-tissue expression
GO: Gene ontology
HR: Hazard ratio
HNSC: Head and neck squamous cell carcinoma
KICH: Kidney chromophobe
KIRC: Kidney renal clear cell carcinoma
KIRP: Kidney renal papillary cell carcinoma
LUAD: Lung adenocarcinoma
LUSC: Lung squamous cell carcinoma
LIHC: Liver hepatocellular carcinoma
LPA: Lysophosphatidic acid
NSCLC: Non-small-cell lung cancer cells
OS: Overall survive
PFS: Progression-free survival
PPS: Postprogression survival
RFS: Relapse-free survival
PKB: Protein kinase B
RAGE: Receptor of advanced glycation end products
STAD: Stomach adenocarcinoma
TP53: Tumor protein 53
TME: Tumor microenvironment
TIMER: Tumor immune estimation resource
THCA: Thyroid carcinoma
TCGA: The Cancer Genome Atlas
TILs: Tumor-infiltrating lymphocytes.
